# Association between screen time and physical activity on mental health among preschoolers: a cross-sectional study from Southwest China

**DOI:** 10.1186/s12889-024-17722-8

**Published:** 2024-01-22

**Authors:** Yi Liang, Qiyun Jin, Junjie Miao, Xiaorong Ni, Xiaoxiao Qian, Yi Xiong, Zhijun Liu, Hongmei Xue

**Affiliations:** 1 Department of Clinical Nutrition, Affiliated Hospital of Guizhou Medical University, Guiyang, Guizhou China; 2 Department of Child Healthcare, Zunyi Maternal and Child Health Care Hospital, Zunyi, Guizhou China; 3 School of Public Health, Hebei Medical University, Shijiazhuang, China; 4 School of Public Health, Guizhou Medical University, Guiyang, Guizhou China; 5 Department of Applied Psychology, School of Management, Zunyi Medical University, Zunyi, Guizhou China; 6 Department of Clinical Nutrition, The First Hospital of Hebei Medical University, Shijiazhuang, Hebei China; 7 College of Public Health, Hebei University, Baoding, Hebei China

**Keywords:** Mental health, Physical activity, Screen time, Strengths and difficulties Questionnaire, Preschoolers

## Abstract

**Background:**

Screen time and physical activity behaviors undergo development during early childhood and impact mental health. However, there is limited knowledge regarding the associations between physical activity, screen time, and mental health problems (MHP) in preschoolers. This study examines these associations using a large sample size and brief measures.

**Methods:**

A multistage cluster stratified sampling method was used to conduct an observational cross-sectional study of 19,015 Chinese preschoolers in 2020. Information on physical activity, and screen time was collected by a self-administered questionnaire; MHP was assessed by the parent-reported Strengths and Difficulties Questionnaire (SDQ). Logistic regression models were used to obtain the odds ratios (ORs) and 95% confidence intervals (95% CIs) of preschoolers’ MHP associated with screen time, total physical activities, moderate to vigorous physical activity (MVPA), and outdoor physical activities.

**Results:**

A total of 19,015 participants from the 19,548 recruited population were included in the analyses (missing rate: 2.73%), 52.60% were boys. 64.01%, 57.96%, 35.98%, and 82.64% of preschoolers were reported to meet total physical activities, MVPA, and outdoor activities with screen time recommendations level. The results of multivariable-adjusted ORs (95% CIs) of preschoolers’ MHP for comparisons of different levels of screen time (< 2 h/day, 2–4 h/day,≥4 h/day) show that screen time positively associated with MHP after adjusting for confounders (*P* < 0.05), but the association was not significant among girls with screen time ≥ 4 h/day. In addition, increased engagement in physical activity was reversely linked to MHP (*P* < 0.05). A stronger association between MHP and MVPA was observed in boys, however, this association was weakened when the total time spent engaging in MVPA exceeded two hours per day (*P* < 0.05).

**Conclusion:**

Less physical activity and more screen time positively relate to MHP, but the relationship differs by type of physical activity, total time, and gender. These findings provide novel insights and evidence supporting for guidelines on physical activity, screen time, and improvement of mental health for preschoolers.

**Supplementary Information:**

The online version contains supplementary material available at 10.1186/s12889-024-17722-8.

## Introduction

Mental health is a state of emotional, behavioral, and social well-being. The most common psychological health problems in early childhood are depression, anxiety, hyperactivity, and difficulties in social interaction [1]. According to statistics, 10–20% of children and adolescents worldwide suffer from mental health problems (MHP) to different degrees [2, 3]. A systematic review demonstrated that MHP was identified in 17.6% of preschoolers, with psychiatric diagnoses reported in 18.4% of this age group [4]. In China, a population-based survey revealed that 17.5% of individuals exhibited MHP [5].

Furthermore, research suggests that preschoolers with MHP may experience a range of adverse health outcomes. These may encompass an increased risk of developing chronic physical conditions such as cardiovascular disease, obesity, and compromised immune function [6]. Additionally, children grappling with MHP may face hurdles in their social and emotional development, significantly influencing their relationships, self-esteem, and overall quality of life [4]. Moreover, untreated MHP in early childhood can have long-term consequences on academic achievement, social adaptation, and psychological well-being [7]. Conversely, positive mental health indicators are beneficially associated with children’s physical, social, intellectual, and emotional development, potentially leaving lasting effects on adolescents and adults [8–10].

Physical activity (PA) and screen time (ST) are separate yet interrelated health-related behaviors that significantly influence children’s MHP. Several reviews found that high levels of PA and low levels of ST were negatively associated with MHP [11–13]. Likewise, two studies conducted in China identified PA as an influential protective factor for children’s development of mental health during the COVID-19 pandemic [14, 15]. However, the PA, ST, and MHP relationships differed between preschoolers and other age groups. For instance, a study focusing on preschoolers indicated no significant association between PA and MHP [16].

Conversely, high levels of ST were found to be negatively linked to prosocial behavior and positively associated with hyperactivity, peer problems, and conduct problems [17]. However, a prospective population-based study revealed a relatively weaker correlation between ST and MHP among preschoolers than among adolescents [18]. These results indicated that the relationship between PA, ST, and MHP in preschoolers remains uncertain. Moreover, it is crucial to simultaneously investigate PA and ST to assess their independent and interactive effects on MHP [19, 20], but a limited number of studies have examined their association [21]. In addition, current research primarily focuses on specific MHP, such as depression, ADHD, and attention deficit-specific mechanisms [22–24]. There remains a dearth of research on the influence of PA and ST on comprehensive MHP.

In general, it is essential to clarify these issues due to the plasticity of health-related behaviors during early childhood, which can have profound implications for subsequent mental health outcomes. Our objective is to examine the associations between different of type PA ST, and MHP among Chinese preschoolers.

## Methods

### Participants and sampling design

The present study originates from the baseline data of the Zunyi Children’s Health Cohort. Preschoolers are children who have not commenced formal preschool or primary education, typically aged between 3 and 6 years [25]. Considering that certain 7-year-old children still have preschool education in China, we included them in the study. Criteria for participation encompass (a) enrollment in the kindergarten of Zunyi City; (b) age ranging from 3 to 7 years; and (c) consent from guardians. Exclusion criteria included a)children with chronic illnesses or prolonged physical health issues; b) children currently undergoing specific medication treatments; c) children with severe behavioral/emotional issues; and (d) congenital intellectual/physical disabilities.

**Fig. 1:**
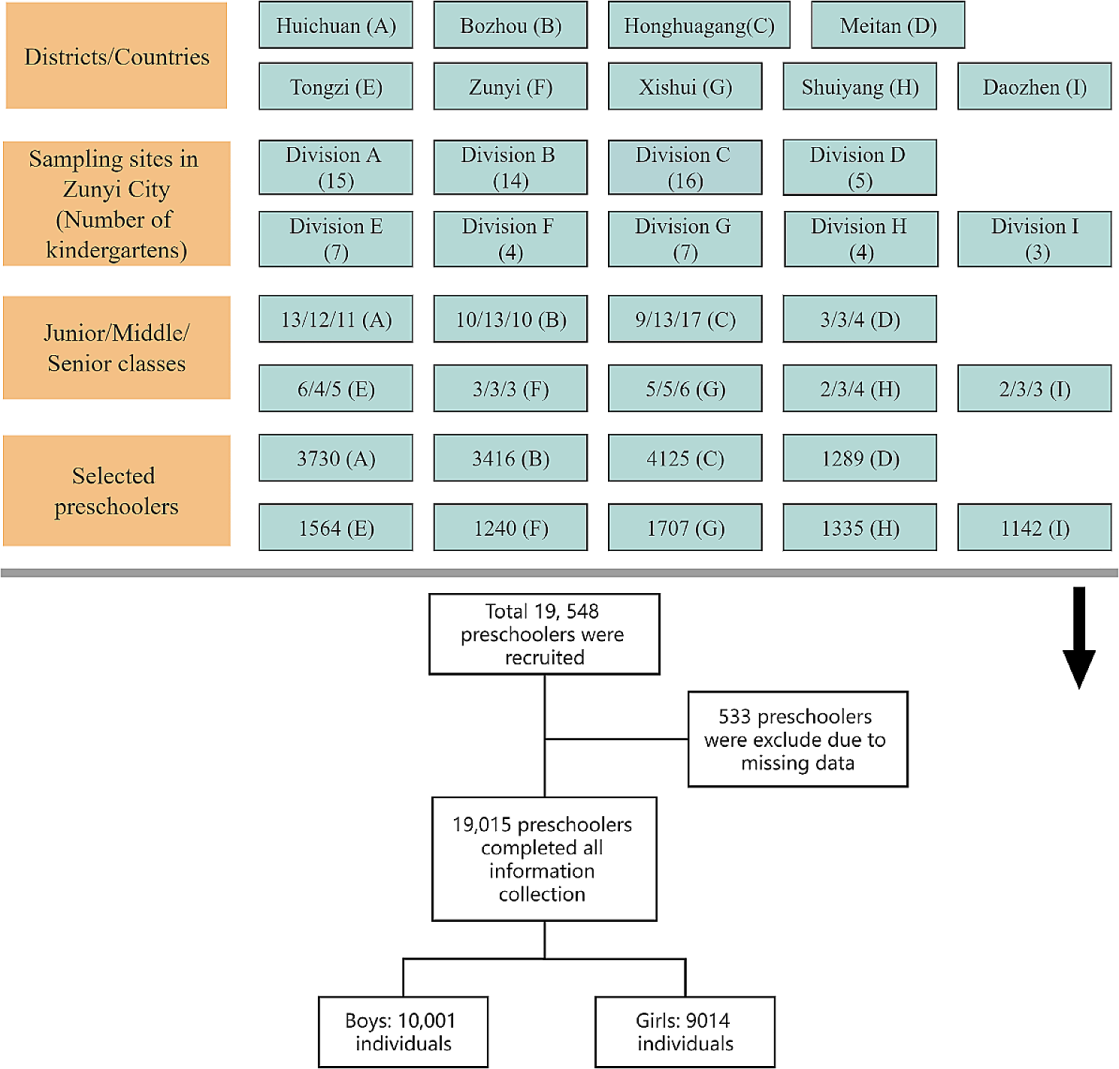
Sampling procedure and participant numbers

This study employed a multi-stage cluster stratified sampling method to select participants (Fig. 1). Participants were from Zunyi City in southwest China, comprising three districts and six county-level administrative centers. We applied simple random sampling to choose three districts and six counties as primary sampling locations, considering geographical zoning, economic development, and urban-rural factors. The sampling locations included the central (Zunyi, Huichuan, Suiyang), southern (Bozhou, Meitan, Honghuagang), northwest (Xishui, Tongzi), and northern (Daozhen, minority autonomous county) parts of Zunyi City. To account for economic factors, we categorized the economic status of each administrative region in Zunyi City based on China’s official data for regional gross domestic product (low-income areas < 9.472 billion CNY; middle-income areas 9.472–36.833 billion CNY; high-income areas ≥ 36.833 billion CNY). Regarding the urban-rural factor, of the nine included sample locations, the ratio of urban to town/rural participants was 0.9: 1.

To determine the sample size, we considered the prevalence of MHP. Reports indicate that the lowest prevalence of MHP in Chinese preschool children is approximately 10.8% [26]. Using the formula:

$$ \frac{{u}^{2}p\left(1-p\right)}{p{\epsilon }^{2}}$$

With a confidence of 95% (two-sided) and a relative error of 15%. We calculated that the average sample size per sampling spot is approximately 1411, accounting for a nonresponse rate of 25%. The sample size was determined to be 15,874 individuals. Simple random sampling was employed to select kindergartens at each sampling spot, with 75 kindergartens ultimately selected as secondary sampling locations for this study. These kindergartens were proportionally allocated to each administrative region. Age was stratified based on the kindergarten class division (junior class: 3–4 years old, middle class: 4–5 years old, senior class: 6–7 years old).

We collected data from 19,548 preschoolers between August and September 2020. Of these, 533 preschoolers were excluded due to incomplete data (missing anthropometric data or information on variables). No missing data imputation or substitution methods were applied. Consequently, the final samples consisted of 19,015 participants, with 10,001 boys (52.6%) and 9,014 girls (47.4%).

Given the participants’ limited reporting ability, all information collected for the present study originated from reports by guardians or caregivers.

### Measures

#### MHP

Preschoolers’ mental health was evaluated using the parent-reported version of the Strengths and Difficulties Questionnaire (SDQ), which is a reliable instrument to screen for MHP among preschoolers [27] and has been validated in China [28]. It contains five subscales (emotional symptoms, conduct problems, hyperactivity/inattention, peer problems, prosocial behaviors) with a total of 25 items; every item was offered three options for respondents, including “0: not true,” “1: somewhat true,” and “2: certainly true”. SDQ total difficulties scores, abbreviated as SDQ scores (range: 0–40), were calculated by summing the scores of four subscales, excluding pro-social behaviors. These scores were utilized to assess the MHP of preschoolers, higher scores indicating a greater degree of MHP. To describe preschooler’s MHP, SDQ scores were divided into three categories following the procedure developed by Goodman [29]: normal range 0–13, borderline range 14–15, and abnormal range 16–40 (Table S1).

#### PA and ST

Validated questionnaires (Cronbach’s alpha 0.74) measured ST and PA, later including total physical activities (TPA), moderate to vigorous physical activity (MVPA), and outdoor physical activities (OPA). The open-ended question was designed to collect information about the total time children spent per day on PA and screen use. ST was accessed by asking guardians or caregivers: “How long the cumulative time did your child spend on electronic devices with a screen (such as mobile phones, TVs, tablets, game machines, etc.) per day?”. Responses were grouped into three categories according to the ST recommendation from the American Academy of Pediatrics [30]: < 2 h/day, 2–4 h/day, and ≥ 4 h/day.

To assess PA, information on TPA, MVPA, and OPA was collected to evaluate preschool children’s activity condition. Regarding measuring TPA, the question posed was “How long the cumulative time did your child spend on total physical activities per day? ”. Similarly, the measuring for MVPA and OPA was illustrated as “How long the cumulative time did your child spend on moderate-to-vigorous activity per day (including cycling, brisk walking, running, swimming, dancing, hiking, etc. )?” and “How long the cumulative time did your child spend on outdoor physical activities per day.” Response for MVPA, and OPA were grouped into three categories according to the recommendations of Physical Activity Guidelines for Chinese Preschoolers aged 3–6 years [25]: <1 h/day, 1–2 h/day, ≥ 2 h/day. For TPA, answers were grouped into three categories: <1 h/day, 1–3 h/day, and ≥ 3 h/day.

### Covariates

Potential covariates were accessed from previous studies [31–35] and collected as a part of questionnaire, which covers fundamental information and sociodemographic characteristics about children and families (age, gender, current height and weight, residence, siblings, gender expectation, average annual household income, parental education level and employment). In addition, another study related to the present study demonstrated a reverse association between the types of weekly food intake and preschooler’s MHP [36]. Consequently, based on the Dietary Guidelines in China, which recommend that preschoolers consume an average of more than 25 foods per week [37], we expanded our investigation into this variable. To gather this information, we inquired with guardians or caregivers whether children consumed more than 25 types of food per week. Considering the link between sleep condition and MHP [38], we collected sleep-related information on preschoolers. Sleep performance was assessed using the Children’s Sleep Habits Questionnaire (CSHQ), and scores beyond 41 indicate sleep disorders [39].

The current height and weight of preschoolers were measured by healthcare practitioners in kindergartens. BMI was calculated as weight divided by height squared [kg/m^2^], and childhood overweight and obesity were defined according to the BMI reference standard of Chinese children and adolescents aged 2–18 years old proposed by Li Hui et al. [40]. Which classified overweight and obesity as BMI greater than or equal to the overweight cutoff point for the same sex and age (in line with adults).

### Data analysis

SAS procedures (version 9.2, SAS Inc., Cary, NC) were used for data analyses. All analyses were performed with a significance level of *p* < 0.05, except for the interaction test, where *p* < 0.1 was considered significant and was entered into logistic regression analysis for adjustment. All continuous variables’ normality was examined using normal probability plots and the Kolmogorov‒Smirnov test. Considering the gender gap in psychological well-being in preschoolers’ growth process, the analysis results in the present study were performed for girls and boys separately [41].

Confounding variables were identified by analyzing the results of covariates and SDQ scores (Table S2), in conjunction with reports from previous studies [32–34]. Binary logistic regression was used to estimate the odds ratios (ORs) and 95% confidence intervals (CIs) of MHP associated with different levels of ST, and PA. In the basic models, ST, TPA, MVPA, OPA, and all covariates were independent predictors. The dependent variables in this study were borderline range (SDQ scores:14–15) and abnormal range (SDQ scores: 16–40); “1” represents abnormal SDQ scores or borderline scores, and “0” represents normal SDQ scores in models.

The following variables potentially affecting these associations were adjusted in Model 1: gender expectation (yes, no), age (years), residence (rural, town, city), siblings (one child, 2–3 children, 4–5 children), parental education level (< 6 years, 6–12 years, and > 12 years of schooling), maternal employment (manual, mental worker), average annual household income (< 6000 CNY, 6000–40,000 CNY, ≥ 40,000 CNY), BMI categories (normal, overweight and obesity), and categories of food per week (< 25 types, ≥ 25 types). Model 2, based on Model 1, additionally adjusted for CSHQ and ST/ TPA.

### Ethics approval and consents to participate

The study followed the principles of the Declaration of Helsinki, and the research procedures were reviewed and approved by the Ethics Committee of Zunyi Maternal and Child Health Hospital (No. 202,110). Parents or legal guardians of the children provided written informed consent to participate in this study.

## Results

### General characteristics

The mean (SD) age of the boys and girls was 4.66 (0.89) and 4.62 (0.87), respectively. Most of children come from sibling families (Table 1). The CSHQ scores for boys and girls were 51.49 (5.84) and 51.47 (5.90), respectively. For boys and girls who consumed more than 25 types of food per week, the number and ratio were 221 (2.21%) and 206 (2.29%). Additionally, we observed a higher prevalence of overweight or obesity among boys than girls (weight: 11.38% vs. 7.84%, obesity: 12.21% vs. 8.20%).

**Table 1:** Participants’ characteristics in the present study ^a^

Group		Boys (*n* = 10,001)	Girls (*n* = 9014)	*P* value
**Child characteristics**				
Age (years)		4.66 ± 0.89	4.62 ± 0.87	0.001
Siblings	One child	2571(25.71)	2043(22.66)	< 0.0001
	2–3 Children	7352 (73.51)	6880 (76.32)	
	4–5 Children	78 (0.78)	91(1.01)	
Resident	Rural	3454 (34.54)	3111 (34.51)	0.30
	Town	1761 (17.61)	1665 (18.47)	
	City	4786 (47.86)	4238 (47.02)	
Body mass index (kg/m^2^)		15.26 ± 3.66	14.79 ± 3.45	< 0.001
BMI categories (%) ^b^	Normal weight	7642 (76.41)	7568 (83.96)	< 0.001
	Overweight	1138 (11.38)	707 (7.84)	
	Obseity	1221 (12.21)	739 (8.20)	
CSHQ score ^c^		51.49 ± 5.84	51.47 ± 5.90	0.80
Food categories per week (≥ 25,%) ^d^		221 (2.21)	206 (2.29)	0.70
**Parental characteristics**				
Gender expectation (yes, %)		2022 (20.21)	2643 (29.32)	< 0.001
Parental high education level (yes, %) ^e^		6786 (67.85)	6107 (67.75)	0.90
Paternal employment (manual, %)		4411 (44.11)	3988 (44.24)	0.80
Maternal employment (manual, %)		3340 (33.40)	2808 (31.15)	< 0.001
Average annual household income ^f^				
	Low	3028 (30.28)	2842 (31.53)	0.001
	Medium	3562 (35.61)	3326 (36.90)	
	High	3411 (34.11)	2846 (31.57)	

^a^ Mean ± SD for normally distributed variables, median (p25, p75) for nonnormally distributed variables, and frequencies for categorical variables. *P* values were calculated from the chi-square test for categorical variables, Kruskal–Wallis tests for nonnormally distributed variables, and variance for normally distributed continuous variables

^b^ Calculated according to China criteria developed by the Capital Institute of Pediatrics

^c^ Calculated based on Children’s Sleep Habits Questionnaire

^d^ According to the Dietary Guidelines in China

^e^ School year ≥ 12

^f^ Low income: <6000 CNY; Medium:6000–40,000 CNY; High:≥40,000 CNY.

### MHP condition and compliance with PA and ST guidelines among preschoolers

For categories of SDQ scores, the percentage of boys with SDQ scores in the borderline or abnormal range exceeds that of girls (borderline: 9.23% vs. 8.55%; abnormal: 9.46% vs. 8.45%, *p* < 0.01)(Table 2). Overall, boys scored higher than girls in SDQ scores, conduct problems, hyperactivity/inattention, and peer problems, but in emotional symptoms and prosocial behaviors, their scores were lower than those of girls. Regarding PA, 12,172 (64.01%) preschoolers reported meeting the recommendation of TPA at least 3 h per day, and 11,022 (57.96%) preschoolers reported engaging in MVPA for at least 1 h per day. In comparison, 6841 (35.98%) preschoolers engaged in OPA for ≥ 2 h per day. Furthermore, the proportion of boys who met the recommended levels of MVPA and OPA according to the guidelines was higher than that of girls (*p* < 0.05). For ST, 15,714 (82.64%) of preschoolers who engaged in ST met the recommendation of less than 2 h per day, with more boys than girls exceeding the 2-hour ST suggestion (*p* < 0.001).

**Table 2:** Conditions of PA, ST, and MHP among preschool children ^a,b^

Measure	Boys (*n* = 10,001)	Girls(*n* = 9014)	*P* value
**PA**			
TPA			
< 1 h/day	812 (8.12)	724 (8.03)	0.90
1–3 h/day	2779 (27.79)	2528 (28.05)	
≥ 3 h/day	6410 (64.09)	5762 (63.92)	
MVPA			
< 1 h/day	4076 (40.76)	3917 (43.45)	< 0.001
1–2 h/day	4106 (41.06)	3666 (40.67)	
≥ 2 h/day	1819 (18.19)	1431 (15.88)	
OPA			
< 1 h/day	1641 (16.41)	1525 (16.92)	0.01
1–2 h/day	4661 (46.61)	4347 (48.22)	
≥ 2 h/day	3699 (36.99)	3142 (34.86)	
**ST**			
< 2 h/day	8122 (81.21)	7592 (84.22)	< 0.001
2–4 h/day	1430 (14.30)	1138 (12.62)	
≥ 4 h/day	449 (4.49)	284 (3.15)	
**MHP**			
SDQ subscales scores			
Total difficulties	10.42 ± 3.74	10.01 ± 3.84	< 0.001
Emotional symptoms	2.12 ± 1.78	2.20 ± 1.84	0.007
Conduct problem	1.33 ± 0.99	1.24 ± 0.97	< 0.001
Hyperactivity/inattention	4.39 ± 1.71	4.06 ± 1.71	< 0.001
Peer problem	2.58 ± 1.10	2.51 ± 1.12	< 0.001
Prosocial behavior	6.44 ± 2.01	6.84 ± 1.97	< 0.001
SDQ scores categories ^c^			
Normal	8132 (82.99)	7481 (82.99)	< 0.01
Borderline	923 (9.23)	771 (8.55)	
Abnormal	946 (9.46)	762 (8.45)	

^a^ Abbreviations: Physical activity, PA; moderate to vigorous physical activities, MVPA; Total physical activities, TPA; outdoor physical activities, OPA; Screen time, ST; Mental health problems, MHP; Strengths and Difficulties Questionnaire; SDQ; Total difficulties scores on the Strengths and Difficulties Questionnaire, SDQ scores

^b^ Mean ± SD for normally distributed variables, and frequencies for categorical variables. *P* values were calculated from the chi-square test for categorical variables, Kruskal–Wallis tests for nonnormally distributed variables, and variance for normally distributed continuous variables

^c^ SDQ scores categories were divided following the procedure developed by Goodman [29]: normal 0–13, borderline 14–15, and abnormal 16–40

### Associations between ST, PA, and MHP among preschoolers

Logistic regression analysis revealed associations between ST, PA, and MHP based on gender (Tables 3 and 4). Multivariable-adjusted ORs (95% CIs) of MHP based on different levels of ST (< 2 h/day, 2–4 h/day, ≥ 4 h) were 1.00, 1.18 (1.02–1.37), 1.82 (1.34–2.44) for boys and 1.00, 1.21 (1.03–1.43), 1.45 (0.95–2.16) for girls, respectively. Our results showed that preschoolers who engage in more ST exhibit higher SDQ scores in both genders ( *p* < 0.05). A slightly stronger relationship was observed in girls at 2–4 h per day of ST. However, when the duration of screen use exceeds 4 h per day, boys show a greater connection to MHP and ST.

**Table 3:** Logistic regression analysis between ST and SDQ scores by gender ^a,b^

	ST (hour/day)				
	< 2		2–4		≥ 4
**Boys (** ***n*** ** = 10,001)**					
**Borderline**					
Unadjusted model	1		1.36 (1.18, 1.57)		1.84 (1.35, 2.48)
Model 1 ^c^	1		1.38 (1.19, 1.59)		1.90 (1.39, 2.56)
Model 2 ^d^	1		1.30 (1.13, 1.51)		1.66 (1.21, 2.26)
**Abnormal**					
Unadjusted model	1		1.18 (1.02, 1.36)		1.96 (1.46, 2.59)
Model 1 ^c^	1		1.21 (1.05, 1.40)		2.03 (1.52, 2.69)
Model 2 ^d^	1		1.18 (1.02, 1.37)		1.82 (1.34, 2.44)
**Girls (** ***n*** ** = 9014)**					
**Borderline**					
Unadjusted model	1		1.13 (0.97, 1.31)		1.59 (1.07, 2.31)
Model 1 ^c^	1		1.14 (0.98, 1.33)		1.63 (1.09, 2.36)
Model 2 ^d^	1		1.04 (0.89, 1.22)		1.36 (0.90, 1.99)
**Abnormal**					
Unadjusted model	1		1.25 (1.08, 1.46)		1.77 (1.19, 2.55)
Model 1 ^c^	1		1.30 (1.12, 1.52)		1.84 (1.24, 2.66)
Model 2 ^d^	1		1.21 (1.03, 1.43)		1.45 (0.95, 2.16)

^a^ Abbreviations: Physical activity, PA; moderate to vigorous physical activities, MVPA; Total physical activities, TPA; outdoor physical activities, OPA; Screen time, ST; Mental health problems, MHP; Strengths and Difficulties Questionnaire; SDQ; Total difficulties scores on the Strengths and Difficulties Questionnaire, SDQ scores

^b^ Association between ST and SDQ scores was analyzed using odds ratios (ORs) and 95% confidence intervals (95% CIs). SDQ scores categories were divided following the procedure developed by Goodman [29]: normal 0–13, borderline 14–15, and abnormal 16–40

^c^ Model 1, adjusted for gender expectation, age, residence, overweight/obesity, siblings, average annual household income, parental education level, maternal employment, and food categories per week

^d^ Model 2, Model 1 additionally adjusted for CSHQ and TPA.

**Table 4:** Logistic regression analysis between PA and SDQ scores by gender ^a,b^

	TPA (hour/day)				MVPA (hour/day)				OPA (hour/day)		
	< 1	1–3	≥ 3		< 1	1–2	≥ 2		< 1	1–2	≥ 2
**Boys (** ***n*** ** = 10,001)**											
**Borderline**											
Unadjusted model	1	0.94 (0.71, 1.25)	0.84 (0.66, 1.08)		1	0.92 (0.80, 1.08)	0.98 (0.81, 1.19)		1	0.81 (0.67, 0.98)	0.89 (0.73, 1.09)
Model 1 ^c^	1	0.96 (0.72, 1.28)	0.85 (0.67, 1.10)		1	0.93 (0.80, 1.08)	0.97 (0.80, 1.17)		1	0.83 (0.68, 1.00)	0.90 (0.74, 1.09)
Model 2 ^d^	1	0.91 (0.68, 1.23)	0.79 (0.62, 1.03)		1	0.93 (079, 1.08)	0.96 (0.79, 1.17)		1	0.81 (0.67, 0.99)	0.88 (0.72, 1.08)
**Abnormal**											
Unadjusted model	1	0.61(0.47, 0.79)	0.56 (0.43, 0.71)		1	0.71 (0.61, 0.82)	0.84 (0.70, 1.01)		1	0.69 (0.58, 0.82)	0.67 (0.55, 0.81)
Model 1 ^c^	1	0.58 (0.45, 0.76)	0.53 (0.42, 0.68)		1	0.71 (0.61, 0.83)	0.81 (0.67, 0.98)		1	0.71 (0.59, 0.85)	0.66 (0.55, 0.80)
Model 2 ^d^	1	0.62 (0.49, 0.80)	0.57 (0.45, 0.73)		1	0.72 (0.61, 0.84)	0.82 (0.67, 0.99)		1	0.69 (0.57, 0.84)	0.65 (0.54, 0.80)
**Girls (** ***n*** ** = 9014)**											
**Borderline**											
Unadjusted model	1	0.92 (0.68, 1.25)	0.79 (0.61, 1.04)		1	0.85 (0.72, 0.99)	0.87 (0.70, 1.08)		1	0.76 (0.62, 0.93)	0.73 (0.59, 0.90)
Model 1 ^c^	1	0.92 (0.68, 1.26)	0.79 (0.61, 1.04)		1	0.86 (0.73, 1.01)	0.87 (0.69, 1.08)		1	0.77 (0.63, 0.94)	0.73 (0.59, 0.90)
Model 2 ^d^	1	0.91 (0.67, 1.26)	0.76 (0.58, 1.01)		1	0.86 (0.73, 1.02)	0.86 (0.69, 1.07)		1	0.78 (0.64, 0.96)	0.73 (0.59, 0.91)
**Abnormal**											
Unadjusted model	1	0.61 (0.47, 0.80)	0.56 (0.44, 0.72)		1	0.74 (0.63, 0.87)	0.84 (0.67, 1.04)		1	0.68 (0.56, 0.82)	0.61 (0.50, 0.75)
Model 1 ^c^	1	0.69 (0.52, 0.91)	0.45 (0.34, 0.59)		1	0.75 (0.64, 0.89)	0.83 (0.66, 1.03)		1	0.71 (0.59, 0.87)	0.62 (0.50, 0.76)
Model 2 ^d^	1	0.68 (0.52, 0.89)	0.45 (0.35, 0.58)		1	0.78 (0.65, 0.92)	0.80 (0.64, 1.01)		1	0.74 (0.61, 0.91)	0.64 (0.51, 0.79)

^a^ Abbreviations: Physical activity, PA; moderate to vigorous physical activities, MVPA; Total physical activities, TPA; outdoor physical activities, OPA; Screen time, ST; Mental health

problems, MHP; Strengths and Difficulties Questionnaire; SDQ; Total difficulties scores on the Strengths and Difficulties Questionnaire, SDQ scores

^b^ Association between PA and SDQ scores was analyzed using odds ratios (ORs) and 95% confidence intervals (95% CIs). SDQ scores categories were divided following the procedure

developed by Goodman [29]: normal 0–13, borderline 14–15, and abnormal 16–40

^c^ Model 1, adjusted for gender expectation, age, residence, overweight/obesity, siblings, average annual household income, parental education level, maternal employment, and food

categories per week

^d^ Model 2, Model 1 additionally adjusted for CSHQ and ST.

Regardless of gender, increased engagement in TPA and OPA was linked to a lower prevalence of MHP (*p* < 0.05). A more pronounced correlation between TPA and MHP was observed in girls, whereas stronger associations with MVPA and OPA were found in boys. Furthermore, we identified a diminished association level between MVPA and MHP when total MVPA time exceeded 2 h per day.

## Discussion

In this large-sample cross-sectional study, we investigated the association between different types of PA (encompassing TPA, MVPA, OPA), ST, and MHP across genders. Results showed reverse associations of high PA, low ST, and MHP after adjusting for potential confounding factors, but these associations varied by type of PA, total time, and gender. Regarding to PA-MHP relationship, Biddle et al. reviewed there were negative associations between PA and indicators of MHP such as anxiety and depression [42]. PA was also reported to relate to better mental outcomes during the COVID-19 Pandemic [43]. In addition, increased TPA was linked to lower emotional and peer problems in a three-year follow-up study [44]. Likewise, OPA proved beneficial for children’s mental health [45], but none of these studies conducted analyses based on gender.

Findings of two prospective analyses suggested higher levels of MVPA were associated with higher hyperactivity and depression/anxiety scores, and these correlations are more significant in boys (46–47), consistent with our study results. Interestingly, we identified a curvilinear association between MVPA and MHP in preschoolers who engaged in high levels of MVPA (≥ 2 h per day) compared to those participating in moderate levels of MVPA (1–2 h per day), and this phenomenon becomes more significant among boys. A study also revealed this curvilinear relationship between MVPA and MHP in adults [48]. It indicated that when adults engage in MVPA for more than 50 min per day, the strength of the inverse association with MHP decreases. Future studies should pay more attention to the dose response between PA and MHP. It should be noted that the measurement of PA in the present study was based on parental or caregiver reports, which poses limitations to the accuracy of PA assessment, especially for MVPA. During MVPA, an individual’s heart rate increases, their breathing accelerates, and multiple muscle groups engage in coordinated activity. Relying on proxy reports for MVPA may not be suitable. However, due to the large sample size of our study, it was impractical to employ objective evaluation methods on participants, such as using devices like accelerometery. Future studies should aim to overcome this limitation. Additionally, some studies have revealed reasons that high levels of PA improve mental health through enhancing social interaction and reducing isolation [49]. Underlying biological mechanisms also demonstrate that PA impacts brain structure and function, including increasing levels of brain-derived neurotrophic factor (BDNF), which can enhance mental health [50].

In contrast to the negative association of PA with MHP, our study revealed excessive ST (≥ 2 h/day) positively linked with MHP among preschoolers, which confirms the findings of a study [51]. It reported that high ST is associated with an increase in emotional and behavioral difficulties, including attention problems and hyperactivity. Another study by Tandon PS et al. also found a positive correlation between ST and higher SDQ scores, indicating an association between ST and a greater MHP [43].

We also observed gender differences in the association between ST and MHP. Specifically, at the same level of ST (2–4 h/day), a slightly stronger association with MHP was observed in girls, these findings were in line with previous research [52, 53]. Whereas boys show a more pronounced connection with MHP when ST exceeds 4 h per day, this differs from Karin’s findings, where Swedish adolescent females rather than males reported increased anxiety when ST exceeded 5 h [47]. Contrastingly, in a follow-up study, Houghton et al. identified a significant association for males, but not for females [54]. Similarly, a review found inconsistent evidence of gender moderating the relationship of ST and psychosocial outcomes among children aged 0–7 years [55]. Explanations for the gender differences on the link of ST and MHP among preschoolers may be related to the type of screen and content although these aspects were not investigated in the present study. A report of a cross-sectional study of UK participants indicated that boys tend to engage more in gaming, while girls are more inclined towards social media usage [56]. Furthermore, the impact of social media and internet use on MHP appears to be greater compared to gaming [57]. Future research on MHP should focus on an analysis of screen usage types and view content.

We cannot ascertain if ST leads to MHP if the reverse is true, or whether other intervening variables impact these correlations in cross-sectional studies. However, an RCT demonstrated that an increase in sedentary behavior per week can significantly elevate anxiety levels compared to a control group [58]. It suggests that reducing sedentary behavior, including screen-related activities, could be a strategy for improving mental health. There’s no universally agreed-upon method for tracking screen behaviors. In the present study, given the participants’ young age, ST reports from parents or caregivers. Hence, the total ST recorded should serve as a proxy of high or low screen usage, rather than a precise measurement. More objective measurement methods are needed to explore the association between ST and MHP in future studies.

Early age is a critical period for children’s brain and physical development, and behaviors of PA and ST during this stage can have a profound impact on the development of cognitive and mental health in children [6, 7]. To our knowledge, this study is the first attempt to examine the association between ST, PA, and MHP among a large sample of preschoolers in China. Notably, we simultaneously assessed preschoolers’ ST and PA levels, considering their potential interactive impact on mental health. Furthermore, the sleep and dietary condition of preschoolers were investigated and adjusted for these as confounding or mediating factors in the present study. A systematic review indicates that currently most studies examine PA, sedentary time, and sleep as separate influences of MHP. However, these factors are interrelated and should be investigated together as a comprehensive behavioral pattern in the future studies [59]. Additionally, many studies adopted a cross-section design, so there is an urgent need for future research to utilize longitudinal cohort designs beginning in the early or toddler years. This is due to the typically unstable behavioral patterns of preschoolers, which contrast with the more consistent and systematic activity patterns observed in adults [60, 61]. Moreover, because of observed differences in links to ST, PA, and MHP, future studies should conduct separate analyses based on sex for these correlations.

Our study also highlights some limitations. We cannot determine a causal relationship between ST, PA, and MHP due to the cross-sectional study design. In addition, although the large sample size and high participation rate led to a diverse group of preschoolers with varying levels of PA and ST, our study did not include information representing physically disabled populations. Therefore, future research should ensure these groups are also included to enhance the representativeness of the findings.

## Conclusions

This study revealed that lower PA and higher ST are linked to MHP in preschoolers. Increased TPA and OPA reversely associatied with MHP, a stronger TPA-MHP link in girls, while a more significant MVPA-MHP association was evident in boys. However, the MVPA-MHP link weakened with over 2 h of MVPA per day. Boys had a more pronounce correlation when ST exceeded 4 h.These results yield insights and the evidence supporting ST and PA guidelines for preschoolers to enhance their mental health.

### Electronic supplementary material

Below is the link to the electronic supplementary material.

**Supplementary Material 1: Table S1.** Bandings of scores of Strengths and Difficulties Questionnaire (SDQ) among preschoolers aged 3–7 years old in the present study ^a^ (n = 19015). **Table S2.** Characteristics of study population stratified by SDQ scores ^a,b^

## Data Availability

All authors agree to share the raw data in the present study without reservation; further acquisition can be directed to Xiaorong Ni.

## Abbreviations

MHP: Mental health problems
MVPA: Moderate to vigorous physical activities
OPA: Outdoor physical activities
PA: Physical activity
SDQ scores: Total difficulties scores on the Strengths and Difficulties Questionnaire
SDQ: Strengths and Difficulties Questionnaire
ST: Screen time
TPA: Total physical activities
