# Supplementary material for: Association between screen time and physical activity on mental health among preschoolers: a cross-sectional study from Southwest China
Source: BMC Public Health. 2024 Jan 22;24:261. doi: 10.1186/s12889-024-17722-8 (PMC10804503; doi:10.1186/s12889-024-17722-8)
Supplement: Supplementary file 1 — Supplementary Material 1: Table S1. Bandings of scores of Strengths and Difficulties Questionnaire (SDQ) among preschoolers aged 3–7 years old in the present study a (n = 19015). Table S2. Characteristics of study population stratified by SDQ scores a,b [file 12889_2024_17722_MOESM1_ESM.docx]

**Table S1.** Bandings of scores of Strengths and Difficulties Questionnaire (SDQ) among preschoolers aged 3-7 years old in the present study **^a^** (n=19015)

| **SDQ scores and subscale** | **Normal range** | | **Borderline range** | | **Abnormal range** | |
| --- | --- | --- | --- | --- | --- | --- |
|  | **Raw data** | **Exact %** | **Raw data** | **Exact %** | **Raw data** | **Exact %** |
| Total difficulties | 0-13 | 82.11 | 14-15 | 8.91% | 16-40 | 8.98 |
| Emotional symptoms | 0-3 | 79.27 | 4 | 9.94 | 5-10 | 10.78 |
| Conduct problems | 0-1 | 73.06 | 2 | 17.06 | 3-8 | 9.88 |
| Hyperactivity inattention | 0-5 | 78.00 | 6 | 12.07 | 7-8 | 9.93 |
| Peer problems | 0-3 | 80.89 | 4 | 16.28 | 5-6 | 2.83 |
| Prosocial behaviors | 10-5 | 87.94 | 4 | 7.10 | 3-0 | 4.95 |

**^a^** Following procedure developed by Goodman (1997) [29]

**Table S2.** Characteristics of study population stratified by SDQ scores **^a,b^**

|  | **Girls (n=9014)** | | | **p value** | **Boys (10001)** | | | **p value** |
| --- | --- | --- | --- | --- | --- | --- | --- | --- |
|  | **Normal** | **Borderline** | **Abnormal** |  | **Normal** | **Borderline** | **Abnormal** |  |
| **Child characteristics** |  |  |  |  |  |  |  |  |
| n (%) | 7481 (47.92) | 771 (45.51) | 762 (44.61) | — | 8132 (52.08) | 923 (54.49) | 946 (55.39) | — |
| Gender expectations (yes, %) | 2282 (30.50) | 189 (24.51) | 172 (22.57) | <0.0001 | 1652 (20.31) | 196 (21.24) | 174 (18.39) | 0.6 |
| Age (years) | 4.61±0.87 | 4.61±0.89 | 4.70±0.86 | 0.03 | 4.65±0.90 | 4.70±0.88 | 4.67±0.88 | 0.3 |
| Residence (city, %) | 3589 (47.97) | 364 (47.21) | 285 (37.40) | <0.0001 | 3977 (48.91) | 429 (46.48) | 380 (40.17) | <0.0001 |
| Body mass index (kg/m^2^) | 14.77±3.43 | 14.73±3.18 | 15.03±3.87 | 0.7 | 15.25±3.58 | 15.29±3.88 | 15.32±4.06 | 0.8 |
| Overweight/obesity **^c^** (%) | 1165 (15.57) | 130 (16.86) | 151 (19.82) | 0.008 | 1930 (23.73) | 203 (21.99) | 226 (23.89) | 0.5 |
| One-child Family (%) | 1663 (22.23) | 204 (26.46) | 176 (23.10) | 0.02 | 2054 (25.26) | 256 (27.74) | 261 (27.59) | 0.3 |
| **CSHQ ^d^** | 51 (47, 54) | 54 (50, 58) | 56 (51, 60) | <0.0001 | 51 (47, 54) | 54 (50, 58) | 55 (50, 59) | <0.0001 |
| **Family characteristics** |  |  |  |  |  |  |  |  |
| Average annual household income **^e^** (%) | 2423 (32.39) | 225 (29.18) | 198 (25.98) | <0.0001 | 2831 (34.81) | 302 (32.72) | 278 (29.39) | 0.0009 |
| High education level **^f^** (%) | 4145 (55.41) | 368 (47.73) | 324 (42.52) | <0.0001 | 4547 (55.91) | 450 (48.75) | 439 (46.41) | <0.0001 |
| Maternal employment (manual, %) | 2322 (31.04) | 234 (30.35) | 252 (33.07) | <0.0001 | 2703 (33.24) | 313 (33.91) | 324 (34.25) | <0.0001 |
| Paternal employment (manual, %) | 3232 (43.20) | 377 (48.90) | 379 (49.74) | 0.5 | 3495 (42.98) | 442 (47.89) | 474 (50.11) | 0.8 |
| **Total food categories**  (25 types/week, %) **^g^** | 1558 (20.83) | 140 (18.16) | 114 (14.96) | <0.0001 | 1679 (20.65) | 159 (17.23) | 162 (17.12) | <0.0001 |

**^a^** Values are mean ± SD for normally distributed variables, median (p25, p75) for non-normally distributed variables, or frequencies for categorical variables. P values are calculated from Chi-square test for categorical variables, Kruskal–Wallis tests for non-normally and variance for normally distributed continuous variables.

**^b^** Calculated based on Total difficulties score of Strengths and Difficulties Questionnaire[29]

**^c^** Calculated according to China criteria developed by Capital Institute of Pediatrics[40]

**^d^** Calculated based on the Children’s Sleep Habits Questionnaire[39].

**^e^** Low income: <6000 CNY; Medium: 6000-40000 CNY; High: ≥40000 CNY

**^f^** School year ≥12.

**^g^** According to Dietary Guideline in China[37]
